# Novel agents in the treatment of multiple myeloma: a review about the future

**DOI:** 10.1186/s13045-016-0282-1

**Published:** 2016-06-30

**Authors:** Leonard Naymagon, Maher Abdul-Hay

**Affiliations:** Department of Medicine, New York University, New York, USA; Perlmutter Cancer Center, New York University, New York, USA; NYU School of Medicine, 240 East 38th Street, 19 Floor, New York, NY 10016 USA

**Keywords:** Multiple myeloma, Novel agents, Immunomodulators, Proteasome inhibitors, Alkylating agents, AKT inhibitors, BTK inhibitors, CDK inhibitors, HDACIs, IL-6 inhibitors, Kinesin spindle protein inhibitors, Monoclonal antibodies, PI3K inhibitors

## Abstract

Multiple myeloma (MM) is a disease that affects plasma cells and can lead to devastating clinical features such as anemia, lytic bone lesions, hypercalcemia, and renal disease. An enhanced understanding of MM disease mechanisms has led to new more targeted treatments. There is now a plethora of treatments available for MM. In this review article, our aim is to discuss many of the novel agents that are being studied or have recently been approved for the treatment of MM. These agents include the following: immunomodulators (pomalidomide), proteasome inhibitors (carfilzomib, marizomib, ixazomib, oprozomib), alkylating agents (bendamustine), AKT inhibitors (afuresertib), BTK inhibitors (ibrutinib), CDK inhibitors (dinaciclib), histone deacetylase inhibitors (panobinostat, rocilinostat, vorinostat), IL-6 inhibitors (siltuximab), kinesin spindle protein inhibitors (filanesib), monoclonal antibodies (daratumumab, elotuzumab, indatuximab, SAR650984), and phosphoinositide 3-kinase (PI3K) inhibitors.

## Background

Multiple myeloma (MM) is the second most common hematologic malignancy and accounts for as many as 20 % of deaths from hematological malignancies and 2 % of deaths from all cancers. In 2012, there were an estimated 89,658 people living with myeloma in the USA. Approximately 0.7 % of men and women will be diagnosed with myeloma during their lifetime, based on the 2010–2012 data. The median age at diagnosis is 65 years, and 5-year survival is 46.6 % [1]. MM may result from the generation and proliferation of malignant plasma cell clones from germinal center lymphocytes, a process that is driven by multiple factors including interleukin 6 (IL-6) and tumor necrosis factor (TNF) alpha. In some instances, MM is a consequence of the malignant transformation of post-germinal center plasma cells, via a proposed two-step model of progression [2]. In the first step, an abnormal response to antigenic stimulation foments limited clonal proliferation and precipitates the premalignant entity of monoclonal gammopathy of undetermined significance (MGUS). A “second hit,” such as dysregulation of cell cycle controls, escapes from normal apoptotic pathways, or a change in the stromal microenvironment, then stimulates the malignant clonal proliferation which characterizes MM. Upon its initial transformation from MGUS, MM often enters a quiescent, or “smoldering,” phase characterized by a relatively measured rate of clonal expansion and the absence of overt clinical symptoms [3]. As the clonal burden becomes substantial, however, dysfunctional plasma cells both directly infiltrate organs and cause indirect damage via the mass production of monoclonal light chains. The resulting outcome is characterized by its wide-ranging and manifold presentations including, but not limited to, anemia, renal failure, bony involvement, hypercalcemia, weight loss, fatigue, and any combination therein [4]. MM is a heterogeneous disease, with its wide spectrum of aggression and treatment resistance likely the result of the various genetic errors and a diverse array of malignant cellular malfunctions, which drive individual clones [5]. Whereas some patients may live a decade or more following diagnosis, others suffer rapid treatment resistant progression and die within 24 months. In spite of recent progress in the development of new and increasingly effective agents, MM remains an incurable disease, which in its end stages is characterized by rapid relapse and broad treatment refractoriness [6, 7].

The past decade has seen extraordinary advances in the treatment of symptomatic MM, particularly with the advent of proteasome inhibitors (such as bortezomib) and immunomodulatory agents (such as lenalidomide), which have become the pillars of frontline treatment regimens [8]. Newly symptomatic patients generally respond well to their first line of treatment and enter a period of remission characterized by stable and effective control of symptoms. As there is no curative treatment, MM inevitably relapses, though it does respond to additional lines of frontline treatment approximately 50 % of the time [7]. Subsequent relapses then occur with increasing frequency and become increasingly refractory to frontline agents. It is during that phase of disease that novel investigational agents enter clinical use as part of clinical trials [9]. Initial treatment strategies depend on the patient’s ability to tolerate intensive treatment. Younger patients (typically those younger than 65) with relatively little comorbidity are treated with high-dose chemotherapy and autologous stem cell transplant (ASCT), whereas older patients, with more formidable comorbidities, receive more moderately dosed chemotherapy only [10]. A decade ago, vincristine-doxorubicin-dexamethasone (VAD) was among the foremost induction regimens; however, it has since been supplanted by bortezomib- and lenalidomide-based regimens, which offer markedly improved response rates at comparable toxicity. Three drug regimens featuring bortezomib, dexamethasone, and an additional agent (typically cyclophosphamide or lenalidomide) are now the standard of care prior to ASCT [11]. The standard conditioning regimen for ASCT is currently melphalan based (Mel200) [8]. A number of trials are presently ongoing to assess the effectiveness of post-ASCT consolidation regimens and establish optimal consolidation standards; however, consensus exists that consolidation therapies should remain brief, with the intent to deepen response while minimizing added toxicity. Following induction, transplant, and consolidation, maintenance therapy is pursued with the goal of prolonging response, delaying progression, and improving overall survival. However, the use of frontline agents in each of these treatment stages has resulted in 5-year survival rates as high as 80 % [8]. Nevertheless, in the absence of a true cure, malignant plasma cell clones do, over time, become increasingly aggressive and increasingly refractory to even frontline treatments, prompting relapse, progression, and death. It is in the arena of such relapsed and refractory disease that novel agents enter into investigational use [12].

Clinical trials, both completed and ongoing, point to an emerging generation of agents which are active in relapsed and refractory myeloma and which may someday form part of an expanded front line. Indeed, some among these novel agents (including pomalidomide, carfilzomib, ixazomib, daratumumab, elotuzumab, and panobinostat) have already been granted FDA approval in the relapsed/refractory setting [9]. The forthcoming generation of therapies will include proteasome inhibitors (marizomib and oprozomib); histone deacetylase inhibitors; kinesin spindle protein inhibitors; and inhibitors of cyclin-dependent kinase (CDK), IL-6, Bruton’s tyrosine kinase (BTK), B cell lymphoma 2 (Bcl-2), protein kinase B (AKT), and phosphoinositide 3-kinase (PI3K) pathways in addition to array of monoclonal antibodies and repurposed alkylating agents. These agents are designed based on our breadth of knowledge regarding malignant plasma cell transformation, proliferation, survival, and clonal expansion. At present, their development statuses run the gamut from preclinical studies to phase 3 trials, to approved clinical use in the relapsed/refractory setting [8, 12].

## Pathophysiology

The sentinel events in the oncogenesis of a malignant plasma cell clone take place in the germinal center, most likely during the mutation-prone processes of isotype class switching and somatic hypermutation [13]. Although these initial mutations may generate a malignant clone, they are typically regarded as necessary but not sufficient for myeloma oncogenesis. The next pathogenic events, where the original clone terminally differentiates into a malignant plasma cell, are thought to take place in the bone marrow [14]. Indeed, the bone marrow microenvironment has been proposed as a key determinant of the progression from pre-myeloma states to malignant disease [15]. The bone marrow niche has been demonstrated to encourage tumor proliferation, resistance to apoptosis, and cancer cell trafficking. In this niche, the premalignant clone takes part in the array of cytokine-mediated cross talk which characterizes the bone marrow milieu and includes a diverse set of resident cells including bone marrow stem cells, mesenchymal stem cells, osteoblasts, osteoclasts, vascular endothelial cells, fibroblasts, adipocytes, monocytes, T cells, and NK cells. The cytokines and soluble factors generated in this neoplastic microenvironment promote clonal proliferation and downregulate apoptotic pathways, while providing greater opportunity for additional oncologically potentiating mutations. Soluble factors generated in the bone marrow niche which have been shown to induce and promote malignant transformation and proliferation include insulin growth factor 1 (IGF-1), IL-6, IL-12, IL-15, Wnt3A, platelet-derived growth factor receptor (PDGFR), vascular endothelial growth factor (VEGF), TNF-α, and numerous others [2, 5, 16]. Among the most studied of these cytokines is IL-6, which is chiefly produced by bone marrow stem cells and macrophages and is an important mediator of myeloma cell growth, survival, migration, and drug resistance [17]. IL-6 is essential to the survival and propagation of both normal and pathologic plasma cells and has been shown to be a required factor for myeloma clones [18]. Myeloma cell lines have been found to have increase expression of the IL-6 receptor, and inhibition of IL-6 has been found to impede myeloma growth [19]. IL-6 is a product of bone marrow stromal cells and acts as a paracrine stimulus for plasma cells. Plasma cell adhesion to bone marrow stroma has been shown to increase stromal IL-6 secretion, thus leading to a self-augmenting feedback loop and demonstrating how amplification of IL-6 pathways may be central to plasma cell tumorigenesis [20]. Indeed, IL-6 has been shown to be a powerful promoter of plasma cell survival and inhibitor of plasma cell apoptosis via numerous pathways including upregulation of Bcl-xL and Mcl-1 [21]. Other cytokines in the bone marrow microenvironment may promote myeloma survival and growth via promotion of local angiogenesis (upregulation of VEGF), evasion of cell-mediated immunity (downregulation of TNF-α and IL-12), promotion of clonal proliferation, and escape from apoptotic pathways (upregulation of PDGF and IGF-1) [2, 5, 12, 16]. The cumulative effect of this cytokine environment is to alter the balance of pro-apoptotic and anti-apoptotic forces within the nascent myeloma cells, favoring unregulated clonal expansion and malignant proliferation. These cytokines and soluble factors all provide potential rational targets for drug design.

## Classifications

Two major systems exist for staging MM: the International Staging System (ISS) and the Durie-Salmon staging system. The ISS is the preferred system due to its simplicity and objectivity. In the ISS, patients are stratified into three categories based on their serum beta-2-microglobulin and albumin levels. Stage I disease is defined as a B2M <3.5 mg/L and serum albumin ≥3.5 g/dL. Stage III disease is defined as a B2M ≥5.5 mg/L. Stage II disease is defined as meeting the requirements for neither stage I nor stage III disease [22].

## Prognostic factors

Prognosis for patients with myeloma is contingent upon multiple factors, some of which are patient specific (age, performance status, comorbidities) and some of which relate to the specific genetic and cellular characteristics of the plasma cell clone. Methods of genetic and cellular phenotyping may be used to stratify myeloma into various risk levels based on the presence or absence of a number of known clone-specific features. Common findings on FISH and karyotyping include t(11;14), t(6;14), t(4;14), t(14;16), t(14;20), del 17p13, trisomies of odd-numbered chromosomes, deletions of chromosome 13, and hypodiploidy. In general, the detection of trisomies portends a better prognosis, and such patients are termed “low-risk.” In general, t(14;16), t(14;20), or del 17p13 portend a poorer prognosis and are regarded to be “high-risk.” These patients account for approximately 15 % of cases and have a median survival of 2 to 3 years with standard treatment. Patients with t(4;14), deletion 13, or hypodiploidy have “intermediate-risk” disease. Patients who lack any of the above abnormalities are “standard-risk” disease [23]. There are a number of other significant characteristics to guide us in establishing the prognosis of MM patients. For instance, high levels of serum beta-2 microglobulin are associated with greater tumor burden and—therefore—poorer prognosis. Plasma cell clones that are more immunophenotypically similar to normal reactive plasma cells likely suggest a better prognosis compared to those with a more abnormal immunophenotype [24]. Similarly, a more abnormal free-light-chain (FLC) ratio may confer a poorer prognosis as may the specific type of monoclonal protein produced [25].

## Established treatments

Patients eligible for ASCT are treated with induction therapy for up to 4 months before stem cell harvesting. Regimens for induction for standard-risk patients include proteasome inhibitor-based regimens such as cyclophosphamide/bortezomib/dexamethasone (CyBorD) and immunomodulator-based regimens such as lenalidomide/bortezomib/dexamethasone (RVD) [26, 27]. The decision between proteasome inhibitor and immunomodulator-based regimens is driven by specific drug availability, patient comorbidities, cost, and patient preference [26]. Patients ineligible for ASCT should still be evaluated for induction with lenalidomide, bortezomib, or an alkylating agent-based regimen [28, 29]. Patients with high-risk disease are often enrolled in clinical trials at the outset of treatment, as prognosis is poor with conventional regimens. High-risk patients unable to enroll in trials often receive induction regimens, which feature both a proteasome inhibitor and immunomodulator such as RVD. Eligible patients then continue on to ASCT with bortezomib-based or lenalidomide maintenance therapy thereafter at times [30, 31]. Patients are evaluated for response following each treatment cycle. A small number of patients will prove refractory to initial treatment, and majority of the patients will eventually relapse following initial treatment. ASCT-eligible patients who did not receive ASCT with initial treatment should be treated with high-dose chemotherapy followed by ASCT at the time of relapse. Patients having already undergone ASCT and demonstrated a significant response may be treated with repeat ASCT or chemotherapy alone at the time of relapse [32]. MM that relapses more than 1 year after initial treatment will typically respond well to a repeated course of the same initial treatment. If relapse occurs sooner, a different treatment regimen will be required [33]. Specific regimens for relapsed and refractory disease are based on available agents such as bortezomib, thalidomide, lenalidomide, carfilzomib, pomalidomide, alkylating agents, anthracyclines, and corticosteroids, administered alone, or more often as components of two or three agent combinations. Patients with MM relapsed or refractory to multiple frontline therapies should be considered for enrollment in clinical trials [7, 8, 12].

## Novel agents

### Immunomodulators

#### Pomalidomide

Pomalidomide is a novel anti-myeloma agent that belongs to the immunomodulatory class. Pomalidomide acts both on myeloma cells and their stromal support systems in the bone marrow microenvironment, to inhibit both intracellular and extracellular myeloma growth mediators. Pomalidomide exerts its immunomodulatory effects by priming natural killer cells and constraining regulatory T cells, thus weakening immune tolerance of myeloma cells and spurring the cellular immune response against them. The effects of pomalidomide may be partially mediated by cereblon, a protein involved in intracellular ubiquitination pathways. Preclinical studies demonstrated that pomalidomide was active against lenalidomide- and bortezomib-resistant cell lines and that its effects were synergistic with dexamethasone [36].

Pomalidomide has been studied extensively and demonstrated impressive results in the treatment of relapsed and refractory MM. In the first such phase 2 trial, 60 relapsed/refractory patients were treated with pomalidomide and low-dose dexamethasone, with 63 % of the patients achieving a confirmed response. Responses were seen in 40 % of the lenalidomide refractory patients, 37 % of the thalidomide refractory patients, 60 % of the bortezomib refractory patients, and 74 % of the patients with high-risk cytogenetic or molecular markers. Median progression-free survival was 11.6 months [37]. In a trial investigating pomalidomide and low-dose dexamethasone in specifically lenalidomide refractory patients, a cohort of 34 such patients demonstrated an overall response rate of 47 % with a median overall survival of 13.9 months [38]. In another phase 2 study, 84 patients refractory to both lenalidomide and bortezomib were enrolled (with a median of 5 prior lines of treatment) and showed a 35 % overall response to pomalidomide and dexamethasone with a median overall survival of 14.9 months and 44 % survival at 18 months [39]. Studies investigating the efficacy of pomalidomide with and without dexamethasone as well as pomalidomide with high-dose and low-dose dexamethasone have demonstrated superior results with pomalidomide and low-dose dexamethasone [40, 41].

Pomalidomide has demonstrated a relatively tolerable safety profile with the most common grade 3/4 toxicities being hematologic (neutropenia, anemia, and pancytopenia) and infectious events. Pomalidomide has demonstrated an increased risk for venous thromboembolism, and concurrent use of VTE prophylaxis has been recommended [41]. In February 2013, pomalidomide was approved by the FDA, for use alone or in combination with dexamethasone, in relapsed/refractory MM patients who have received at least two prior therapies including lenalidomide and bortezomib and have demonstrated disease progression within 60 days of their most recent treatment. Trials are in progress, which combine pomalidomide/dexamethasone with various other agents including cyclophosphamide, clarithromycin, pegylated liposomal doxorubicin, and proteasome inhibitors [42].

### Proteasome inhibitors

#### Carfilzomib

The proteasome is the ultimate destination for ubiquitinated proteins marked for degradation and clearance from the intracellular space. In this way the proteasome acts as a regulator of cytoplasmic protein expression. Proteasome inhibition prompts the accumulation of misfolded and ubiquitinated intracellular debris and prevents the degradation of pro-apoptotic factors, thus promoting programmed cell death. Malignant cells, which depend heavily on the suppression of apoptotic pathways, are particularly sensitive to this interruption of routine proteolysis. The proteasome has also been shown to regulate intracellular levels of the anti-apoptotic protein NF-kB that is constitutively present in the cytosol and inactivated by the IkB family inhibitors. When phosphorylated, IkB is targeted for degradation by the 26S proteasome, allowing translocation of NF-kB into the nucleus. Proteasome inhibition increases the availability of IkB within the cytosol, thus inhibiting NF-kB and impairing one of the anti-apoptotic mechanisms of NF-kB-dependent tumor clones [43–45].

Bortezomib, the forerunner of its class and potent inhibitor of the 26S proteasome, has become a frontline agent in the treatment of MM since its approval by the FDA in 2003. Carfilzomib, the most prominent of the novel proteasome inhibitors, irreversibly binds the 20S proteasome, preventing its chymotrypsin-like activity and promoting the accumulation of pro-apoptotic polyubiquitinated proteins, resulting in cell cycle arrest, programmed cell death, and inhibition of tumorigenesis. In vitro studies have demonstrated that carfilzomib is a more specific inhibitor of chymotrypsin-like proteolysis at the proteasome than bortezomib and has fewer off target effects. These preclinical observations have been followed by clinical data demonstrating the relatively more benign toxicity profile of carfilzomib, most significantly its lower association with peripheral neuropathy. Even more encouraging has been the seemingly weak and incomplete cross-resistance between bortezomib and carfilzomib, a promising finding for bortezomib refractory patients [44, 45].

Single-agent carfilzomib has demonstrated significant efficacy in relapsed/refractory MM. Two hundred sixty-six patients, 95 % of whom were refractory to their most recent therapy and 80 % of whom were either refractory to or intolerant to both bortezomib and lenalidomide, demonstrated an overall response rate of 23.7 % with a median duration of response and median overall survival of 7.8 and 15.6 months, respectively. Common adverse events included fatigue (49 %), anemia (46 %), nausea (45 %), and thrombocytopenia (39 %). Only 12.4 % of patients experienced peripheral neuropathy [43]. It was on the strength of this phase 2 study that carfilzomib obtained its initial FDA approval for the treatment of relapsed/refractory myeloma. It is presently approved for use in patients who have received at least two prior therapies, including bortezomib and an immunomodulatory agent, and have demonstrated disease progression within 60 days of completing their most recent therapy [45, 46].

In the ASPIRE trial, 792 patients with relapsed MM were randomized to either carfilzomib with lenalidomide and dexamethasone or to lenalidomide and dexamethasone alone. The addition of carfilzomib was found to significantly improve progression-free survival to 26.3 months in the carfilzomib group versus 17.6 months in the control group (HR = 0.69, CI 0.57 to 0.83, *p* = 0.0001). Overall response rate (ORR) in the carfilzomib group was 87.1 % compared to 66.7 % in the control group, and the complete response (CR) rate with carfilzomib was 31.8 % compared with 9.3 % among controls. The adverse event rate was similar in the two groups; however, patients in the carfilzomib group reported superior health-related quality of life [45].

ENDEAVOR was a phase 3 trial and directly compared bortezomib to carfilzomib in relapsed MM patients. Nine hundred twenty-nine patients, whose disease had relapse after at least one but no more than three prior treatment regimens, were randomized to either carfilzomib with low-dose dexamethasone or bortezomib with low-dose dexamethasone. Based on preliminary analysis of interval outcomes, carfilzomib has demonstrated clear clinical superiority with regard to the primary endpoint of progression-free survival (PFS). Median PFS in the carfilzomib and bortezomib groups at the time of the most recent cutoff were 18.7 and 9.4 months, respectively (HR = 0.53, 95 % CI 0.44–0.65). Furthermore, neuropathy rates were found to be significantly lower in the carfilzomib group. The study results were presented at the American Society of Clinical Oncology 2015 Annual Meeting showing ORRs as 76.9 % in the carfilzomib arm versus 62.6 % (*p* < .0001) in the bortezomib arm. Additionally, 54.3 % (carfilzomib) versus 28.6 % (bortezomib) had a very good partial response (PR) or better, and 12.5 versus 6.2 % of the patients had a complete response or better. Treatment discontinuation due to an adverse event (AE) occurred in 14.0 % in the carfilzomib arm and 15.7 % of the patients in the bortezomib arm. Overall survival data were immature and continue to be followed [46, 47].

Carfilzomib is also being evaluated as a potential induction agent for newly diagnosed MM. In a phase 2 trial of carfilzomib with cyclophosphamide and dexamethasone, in elderly patients with newly diagnosed MM, the regimen induced high CR rates and was associated with low toxicity [48]. Fifty-eight patients were treated with carfilzomib/cyclophosphamide/dexamethasone followed by carfilzomib maintenance until progression or intolerance with 95 % of the patients achieving at least a PR (including 71 % with at least a very good partial response (VGPR), 49 % with at least near CR, and 20 % with stringent CR). The most frequent toxicities were neutropenia (20 %) and anemia (11 %) with relatively few cases of mild peripheral neuropathy (9 %). A similar trial examined cyclophosphamide, carfilzomib, thalidomide, and dexamethasone (CYCLONE) in 64 patients with newly diagnosed MM, with 91 % of the patients demonstrating at least a PR and 59 % demonstrating at least a VGPR [49]. Stem cell collection was successful in all patients in whom it was attempted with PFS and OS at 24 months found to be 76 and 96 %, respectively. Carfilzomib has also been demonstrated to be an effective induction agent in combination with melphalan and prednisone among transplant-eligible patients. Toxicities were found to be manageable and peripheral neuropathy rare [50].

#### Ixazomib

Ixazomib is an inhibitor of the 20S proteasome and the first oral proteasome inhibitor to enter clinical trials [51]. Preclinical studies have demonstrated that ixazomib is active in bortezomib-resistant cell lines and that its effects are synergistic with lenalidomide and dexamethasone [51]. A phase 1 trial of ixazomib that enrolled 60 patients with relapsed/refractory MM demonstrated manageable toxicities (thrombocytopenia, diarrhea, nausea, fatigue, and vomiting being the most prominent) with a 20 % incidence of all peripheral neuropathy and 2 % incidence of grade 3 peripheral neuropathy. Eighteen percent of the heavily pretreated cohort achieved PR or better [51]. A similar phase 1 study, which also enrolled 60 patients, demonstrated similar toxicities and a 12 % overall incidence of neuropathy. Fifteen percent of these heavily pretreated patients achieved PR or better with 76 % of patients achieving stable disease or better [52]. A phase 1/2 study investigating the combination of ixazomib, lenalidomide, and dexamethasone in patients with newly diagnosed MM enrolled 65 patients. Fifty-eight percent of the patients were found to have very good PR or better [53]. These results were followed by the TOURMALINE-MM1 phase 3 trial, a randomized, double-blind, placebo controlled clinical study of 722 patients evaluating ixazomib plus lenalidomide and dexamethasone compared to placebo plus lenalidomide and dexamethasone in adult patients with relapsed and/or refractory MM. The study showed a PFS of 20.6 in the ixazomib arm versus 14.7 months in the control arm (*p* = 0.012). ORR was 78.3 % in the ixazomib arm with median duration of response was 20.5 months, versus 71.5 % and 15 months in the control arm. Median PFS in high-risk patients was similar to that in the overall patient population and in standard-risk patients. The most common grade ≥3 adverse events in the ixazomib group included neutropenia, anemia, thrombocytopenia, and pneumonia [54]. The FDA granted ixazomib (trade name Ninlaro) approval in November 2015 based on the result of this study. Ninlaro is approved for use in combination with lenalidomide and dexamethasone to treat MM patients who have received at least one prior therapy.

#### Marizomib

Marizomib is an investigational proteasome inhibitor, which has been shown to irreversibly bind to all three catalytic subunits of the 20S proteasome [55]. Marizomib’s irreversible binding to the 20S proteasome and its significantly lower rate of efflux from malignant cells appear to account for its increased cytotoxicity and longer duration of action, as well as its vigorous activity in bortezomib-resistant cell lines. Phase 1 studies, though limited to date, have demonstrated relatively mild toxicities and no evidence of neuropathy or thrombocytopenia. In a dose escalation study of 15 relapsed/refractory myeloma patients treated with marizomib monotherapy, three patients, all of whom were bortezomib resistant, demonstrated at least PR. Marizomib has demonstrated in vitro synergy with a number of other anti-myeloma agents including immunomodulators and histone deacetylase inhibitors. Furthermore, because marizomib and bortezomib are structurally dissimilar, and influence different apoptotic signaling pathways, there exists strong rationale for using the two agents in combination, especially given in vitro studies demonstrating encouraging synergy [55, 56].

#### Oprozomib

Oprozomib is a structural analog of the 26S proteasome inhibitor carfilzomib, which, unlike carfilzomib, is orally bioavailable [57]. Oprozomib is only 20 % as potent as carfilzomib, however, demonstrates similar cytotoxicity with longer exposure as a result of its time-dependent proteasome inhibition [58]. Phase 1 studies have demonstrated a tolerable safety profile with low incidence of neuropathy [57]. Twenty-nine patients with relapsed/refractory MM were enrolled in a dose escalation study of oprozomib/dexamethasone combination therapy. The primary challenges to tolerability proved gastrointestinal with frequent diarrhea, nausea, and vomiting. However, none of the enrolled patients demonstrated new or worsening of baseline neuropathy [59]. Preliminary response rates in several phase 1 studies of heavily pretreated patients have been encouraging though sample sizes remain small [57, 59, 60].

### Monoclonal antibodies

#### Daratumumab

Daratumumab is a human IgG1k monoclonal antibody against CD38, a cell surface protein that is prominently expressed on myeloma cells and plays numerous roles in myeloma tumorigenesis. CD38 is a regulator of cell adhesion and likely helps mediate a favorable stromal environment for myeloma cells. As a regulator of intracellular calcium signaling, CD38 is involved in the messenger pathways which regulate apoptosis, survival, and proliferation. In addition, CD38 mediates cross talk with B cells, T cells, and NK cells and may thus be a factor in immune tolerance of malignant plasma cells. Finally, binding of daratumumab to CD38 has been shown to mediate phagocytosis of MM cells by macrophages [61, 62].

A phase 1/2 trial investigated the efficacy of daratumumab with lenalidomide among 32 patients with relapsed and refractory MM. The ORR in this heavily pretreated population was found to be 88 %, with VGPR found among 53 % of patients. Neutropenia was the most commonly encountered adverse event occurring among 81 % of patients [63]. After demonstrating promise in combination with lenalidomide, daratumumab was investigated as a single agent in relapsed/refractory myeloma. Seventy-two heavily pretreated patients were enrolled. Thirty patients received scheduled doses of daratumumab at 8 mg/kg while the remaining 42 patients received scheduled doses of 16 mg/kg. Seventy-nine percent of the patients enrolled had disease refractory to their most recent line of treatment, including 64 % of patients refractory to both bortezomib and lenalidomide. The most common grade 3 or 4 adverse events were pneumonia and thrombocytopenia. No dose-limiting toxicities were reported. Daratumumab monotherapy demonstrated encouraging efficacy in this exceptionally refractory population, with better response noted in the 42 patients that received the higher dose (16 mg/kg). The ORR was 36 % in the cohort that received 16 mg/kg (15 patients had PR or better, 2 had VGPR, and 2 had CR) and 10 % in the cohort that received 8 mg/kg (3 patients had PR). In the cohort that received 16 mg/kg, the median PFS was 5.6 months and 65 % of the patients who had a response did not have progression at 12 months [64].

Seeking to build on the promise of the above studies, a phase 2 trial is presently investigating daratumumab monotherapy in MM patients with at least three lines of prior therapy or double refractory disease. One hundred six heavily pretreated (with a median of 5 previous lines of therapy), poly-refractory (95 % refractory to the most recent proteasome inhibitor and immunomodulatory drug used) patients were enrolled at the time of the most recent cutoff. Adverse events were fatigue (39.6 %), anemia (33.0 %), nausea (29.2 %), thrombocytopenia (25.5 %), back pain (22.6 %), neutropenia (22.6 %), and cough (20.8 %). Five patients (4.7 %) discontinued treatment due to adverse events. ORR was 29.2 % with a 7.4-month median duration of response. Median time to progression was 3.7 months. Median overall survival has not been reached and the estimated 1-year OS rate is 65 %. After a median follow-up of 9.4 months, 14 out of 31 (45.2 %) responders remain on therapy [65].

A pooled analysis of 148 patients treated with daratumumab monotherapy at a dose of 16 mg/kg, including those patients in the above trials, lends further support to the agent’s use in relapsed/refractory disease. The pooled population had received a median of 5 prior lines of treatment, and 86.5 % were double refractory. ORR was 31.1 % among this heavily pretreated and extensively refractory population. The median duration of response was 7.6 months, median PFS was 4.0 months, and overall survival was 20.1 months [66]. Given this well-demonstrated efficacy in a population with limited established treatment option, daratumumab is fast making inroads into the MM treatment paradigm. In November 2015, the FDA granted accelerated approval for daratumumab (Darzalex) to treat MM patients who have received at least three prior treatments [67]. More recently, and based on the strength of the above trial data, daratumumab was approved under accelerated assessment by the European Medicines Agency for treatment of MM patients who are refractory to both proteasome inhibitors and immunomodulatory agents. Along with elotuzumab, daratumumab is the first monoclonal antibody approved for the treatment of MM and demonstrates significant promise for treatment of relapsed and refractory disease.

#### Elotuzumab

CS1, a subunit of CD2, is a cell surface glycoprotein and member of the signaling lymphocyte activation molecule (SLAM) family. CS1 is consistently expressed by MM cells and rarely expressed in other tissues including hematopoietic elements. The role of CS1 in the pathogenesis of MM is unclear; however, it remains a rational target for novel therapies. Elotuzumab is a humanized IgG1 monoclonal antibody against CS1. Preclinical studies demonstrated activity against MM which was synergistic with proteasome inhibitors and immunomodulatory agents. Phase 1 trials demonstrated the tolerability of elotuzumab and provided preliminary indications of its clinical efficacy. The most commons adverse event was infusion reaction (present in 27–71 % of patients in phase 1 trials) which was typically preventable with premedication prior to infusion. The most common grade 3/4 adverse events were hematologic, most often lymphopenia [68].

While a phase 1 trial of elotuzumab monotherapy in 35 relapsed refractory patients demonstrated no objective response, trials with combination therapy have proved more encouraging [107]. A phase 1 study of elotuzumab with bortezomib in 28 relapsed/refractory patients yielded an ORR of 48 %, and a similar study of elotuzumab with lenalidomide yielded an ORR of 82 % [68]. A phase 2 study of 73 relapsed/refractory lenalidomide naïve patients treated with lenalidomide, dexamethasone, and either low- or high-dose elotuzumab yielded an encouraging response as well. ORR was 84 % across all patients, 92 % in the low-dose cohort and 76 % in the high-dose cohort. Median PFS was not reached in the low-dose group and was 18.6 months in the high-dose group, with a median follow-up of 20.8 months [68]. Thus, elotuzumab demonstrated significant efficacy in this relapsed/refractory population, though treatment at the lower dosage proved more effective than treatment at the higher dosage. Elotuzumab in combination with bortezomib and dexamethasone was evaluated among 152 patients with relapsed/refractory MM. Patients were randomized to receive either bortezomib/dexamethasone alone or in combination with elotuzumab. The elotuzumab group demonstrated a median PFS of 9.7 months compared to 6.9 months among the control group, yielding a hazard ratio (HR) of 0.72. VGPR or better occurred in 36 % of patients in the elotuzumab group compared with 27 % in the control group. Addition of elotuzumab did not seem to add clinically significant toxicity to the treatment regimen [69].

Building on the strength of the above early-phase trials, the first phase 3 trial of elotuzumab in MM, the ELOQUENT 2 trial, included 646 relapsed/refractory patients randomized to receive elotuzumab plus lenalidomide/dexamethasone versus lenalidomide/dexamethasone alone. The primary end points were PFS and ORR. At 1 year, PFS in the elotuzumab and the control groups was 68 and 57 %, respectively, and at 2 years, PFS was 41 and 27 % respectively. Median PFS was 19.4 months in the elotuzumab group and 14.8 months in the control group. Addition of elotuzumab to lenalidomide and dexamethasone carried a hazard ratio of 0.70 (CI 0.57 to 0.85, *p* < 0.01) for progression or death. ORR was 79 % in the elotuzumab group and 66 % in the control group (*p* < 0.001) [70]. Accordingly, elotuzumab (Empliciti) was granted FDA approval in November 2015 for relapsed/refractory MM patients in combination with lenalidomide and dexamethasone. Elotuzumab and daratumumab are the first monoclonal antibodies approved for use in MM and herald the rise of immunotherapy to a position of prominence in the myeloma treatment paradigm. An additional phase 3 trial, ELOQUENT 1, comparing elotuzumab with lenalidomide/dexamethasone to lenalidomide/dexamethasone alone among patients with previously untreated MM is currently ongoing.

#### Indatuximab

Indatuximab is a chimerized anti-CD138 monoclonal antibody conjugated to the maytansinoid cytotoxin DM4, a potent inhibitor of the microtubule assembly [71]. CD138 is a relatively exclusive plasma cell marker, with minimal expression among other hematopoietic lineages. CD138 expression is considerably upregulated in MM cells, as well as in other hematologic, solid, and neuroendocrine tumors. Overexpression of CD138 on malignant plasma cells is substantial and makes it among the most specific target antigens for MM. Conjugation of anti-CD138 to DM4 allows for the targeted delivery of cytotoxins to myeloma cells. Indatuximab is internalized at the cell surface, releasing DM4 into the cytoplasm where its anti-tubulin effects promote cell death [71]. Preclinical studies have demonstrated considerable synergy between indatuximab and lenalidomide, prompting the design of a phase 1/2a trial investigating indatuximab-lenalidomide-dexamethasone among relapsed/refractory patients [72]. Fifteen patients were enrolled, of whom 87 % had prior lenalidomide exposure and 50 % were lenalidomide/dexamethasone refractory. The patients were divided into low-, intermediate-, and high-dose groups with respect to indatuximab. The most common adverse events were fatigue, hypokalemia, and diarrhea, and two patients withdrew due to toxicity. ORR was 78 % with all non-responders achieving disease stabilization [72]. The study was insufficiently powered to detect dose dependence but showed the potentials of indatuximab in the treatment of refractory/relapsed MM patients.

#### SAR (SAR650984)

Like daratumumab, SAR650984 is a monoclonal antibody to CD38. SAR650984 exerts anti-tumor activity via antibody-dependent cell-mediated cytotoxicity, complement-dependent cytotoxicity, direct apoptosis induction, and allosteric inhibition of CD38 enzymatic activity [73]. In a phase 1 dose escalation trial, 35 patients with heavily pretreated relapsed myeloma received SAR650984. SAR650984-related adverse events (grade 3/4) included pneumonia (*n* = 3), with hyperglycemia, hypophosphatemia, pyrexia, apnea, fatigue, thrombocytopenia, and lymphopenia in one patient each. ORR was 24 % and CR was 6 %. In the subset of patients treated at higher dose levels (*n* = 18), ORR was 33 % and CR was 11 % without a significant increase in adverse events [73]. An additional phase 1 trial investigated the combination of SAR650984 and lenalidomide in 31 heavily pretreated and relapsed patients. There were no dose-limiting toxicities. The most common adverse events were fatigue (41.9 %), nausea (38.7 %), upper respiratory tract infection (38.7 %), and diarrhea (35.5 %). Infusion-associated reactions occurred in 38.7 % of patients and prompted treatment discontinuation in 2 patients. ORR was 64.5 % and clinical benefit response (CBR) was 71 %. Among patients relapsed and refractory to both immunomodulatory agents and proteasome inhibitors (*n* = 21), ORR was 52.4 % and CBR was 61.9 %. Median PFS among all patients was 6.2 months. Among patients pretreated with proteasome inhibitors or immunomodulators, median PFS was 4.8 months [74].

### Histone deacetylase inhibitors

#### Panobinostat

Histone acetylation and deacetylation plays important roles in the regulation of gene expression [75]. In general, hyper-acetylated chromatin is transcriptionally active, and hypo-acetylated chromatin is transcriptionally silent. Altering the acetylation of chromatin may thus alter the expression of oncogenes and tumor suppressors and thus influence both oncogenesis and play a role in rational drug design. The histone deacetylase (HDAC) 6 has been shown to serve an additional function, as an acetylator and regulator of the aggresome protein degradation pathway. HDAC6 inhibition blocks aggresome formation, thus inhibiting the degradation of misfolded proteins and causing their accumulation within cells. This both echoes and interrelates with the proposed mechanism of proteasome inhibition, which is characterized by the prevention of ubiquitinated protein degradation within the proteasome and explains the observation of synergy between histone deacetylase inhibitors and proteasome inhibitors [75]. Panobinostat is an oral pan-deacetylase inhibitor, which increases the acetylation of proteins involved in numerous oncogenic pathways, including the abovementioned aggresome protein degradation pathway. Preclinical studies demonstrated that inhibition of the aggresome pathway by panobinostat, when combined with inhibition of the proteasome pathway by bortezomib, resulted in synergistic cytotoxicity among MM cells [76].

The PANORAMA 2 trial evaluated the combination of panobinostat and bortezomib along with dexamethasone in patients with relapsed and bortezomib refractory MM. Fifty-five heavily pretreated patients with a median of four prior regimens and two prior bortezomib-containing regimens were enrolled. ORR was found to be 34.5 %; clinical benefit rate was 52.7 % and median PFS was 5.4 months. The most common adverse events were thrombocytopenia (64 %) followed by fatigue and diarrhea [76]. In a phase 3 randomized double blinded study (PANORAMA 1 trial), patients with relapsed or refractory MM received bortezomib, dexamethasone, and either panobinostat or placebo. A total of 768 heavily pretreated patients were randomized in that trial. The primary endpoint, PFS, was 12 months in the panobinostat group and 8.1 months in the placebo group (*p* < .0001; HR 0.63, 95 % CI (0.52, 0.76)). Discontinuation of treatment due to adverse events occurred in 36 % of patients in the panobinostat group and 20 % of patients in the placebo group. Patients in the panobinostat group proved considerably more likely to demonstrate thrombocytopenia (67 vs 31 %), neutropenia (35 vs 11 %), and diarrhea (26 vs 8 %) [77]. Given the strength of the above trials, the FDA approved panobinostat (under the trade name Farydak) in February 2015, in combination with bortezomib and dexamethasone, for treatment of MM patients who have received at least two prior standard therapies (including bortezomib and an immunomodulatory agent) [78].

#### Ricolinostat

Ricolinostat (ACY-1215) is an HDAC6-specific histone deacetylase inhibitor. The combination of ricolinostat with the novel proteasome inhibitor carfilzomib has demonstrated synergistic toxicity to myeloma cells resistant to bortezomib in the preclinical setting [79]. Proteasome inhibition was shown to precipitate the accumulation of misfolded and ubiquitinated proteins within the aggresome, and HDAC6 inhibition was shown to disrupt proper aggresome formation and function [79]. The resulting mass aggregation of ubiquitinated intracellular detritus, with no available mechanism for disposal, prompted activation of apoptotic pathways. Similar synergistic effects have been demonstrated with ricolinostat and other proteasome inhibitors [80]. Murine models of MM have demonstrated significant delay in tumor growth and significant prolongation of survival when treated with combination of ricolinostat and bortezomib [80].

#### Vorinostat

Vorinostat is an orally bioavailable, non-specific histone deacetylase inhibitor. It was approved by the FDA in 2006 for the treatment of cutaneous T cell lymphoma and has shown activity in other hematologic and non-hematologic malignancies as well [81]. Phase 1 studies in patients with MM demonstrated a modest side effect profile and suggested promising activity in combined regimens [82–84]. VANTAGE 095 was a phase 2b trial of vorinostat and bortezomib in bortezomib refractory patients who were either refractory, ineligible, or intolerant to immunomodulator-based regimens [85]. One hundred forty-three heavily pretreated patients, all of whom had received prior treatment with both bortezomib and an immunomodulator, were enrolled. ORR, the primary endpoint, was 17 % in this population of bortezomib refractory patients, with a clinical benefit rate of 31 % and a median duration of response of 6.3 months [86]. VANTAGE 088 was a phase 3 trial comparing bortezomib and vorinostat to bortezomib and placebo in patients with non-refractory MM (patients with known resistance to bortezomib were excluded from the study) [81]. Three hundred seventeen patients were included in the vorinostat group and 320 in the placebo group. PFS was the primary endpoint and was 7.63 months in the vorinostat group versus 6.83 months in the placebo group (*p* = 0.01). Incidence of thrombocytopenia was considerably higher in the vorinostat group (45 vs 24 %) while other toxicities were comparable [81]. Vorinostat may thus be a salvage option in patients that are refractory to bortezomib and immunomodulators; however, its effect in bortezomib-sensitive patients, although significant, is of unclear practical clinical utility [87].

### Alkylating agents

#### Bendamustine

Bendamustine is a well-known alkylating agent initially developed in the 1960s, which has more recently been under increasing investigation for its potential utility in the treatment of MM [88]. Bendamustine acts as a classical alkylating agent of the nitrogen mustard class by inducing cell cycle arrest and promoting apoptosis via the alkylation of DNA [88]. Recent trials have demonstrated the efficacy of bendamustine, in combined regimens, among broader cohorts of myeloma patients, and in particular among those with relapsed and/or refractory disease [89].

The combination of bendamustine, lenalidomide, and dexamethasone (BLD) has been shown to be safe and effective in patients with relapsed or refractory MM. A multicenter phase 1/2 trial with a total of 29 enrolled relapsed/refractory patients treated with BLD demonstrated a PR rate of 52 %, with very good PR achieved in 24 %, and minimal response in an additional 24 % of patients. One-year OS was 93 %, and median PFS was 6.1 months with 1-year PFS of 20 %. Grade 3/4 adverse events were largely hematologic including neutropenia, thrombocytopenia, and anemia [90]. The combination of bendamustine with bortezomib and dexamethasone has also been shown to be an active and well-tolerated regimen in patients with relapsed or refractory myeloma. This combination was evaluated in 79 relapsed refractory patients. The primary endpoint, ORR, was 60.8 %. PFS was 9.7 months and OS was 25.6 months. The most common adverse events were again hematologic [91]. An additional trial of combination bendamustine-bortezomib-dexamethasone, in this instance investigating its use as a second-line treatment among elderly patients at the time of relapse, has also demonstrated encouraging results. A total of 73 (median age 76 years) patients were enrolled with the primary end point being overall response rate (ORR). ORR assessed during treatment was 69.8 %. A total of 57.6 % of patients achieved at least PR. A complete response was seen in 10.9 % of the patients, a very good partial response (VGPR) in 16.5 %, and a PR in 29 39.7 % [92]. Bendamustine has also been investigated, in combination with thalidomide and dexamethasone, as a salvage option in relapsed and/or refractory myeloma patients to both bortezomib and lenalidomide. In a retrospective analysis of 30 such double refractory patients treated with the bendamustine-thalidomide-dexamethasone combination, 87 % achieved stable disease or better. At a median follow-up time of 12.1 months, median PFS and overall survival were 4.0 and 7.2 months, respectively. The most common grade 3–4 adverse events were again hematological toxicities [93]. Numerous phase 1 and 2 clinical trials are now underway to investigate the combination of bendamustine with carfilzomib and dexamethasone in relapsed/refractory patients, as well as the combination of bendamustine, melphalan, and carfilzomib as a preparative regimen prior to autologous stem cell transplant.

### AKT inhibitors

#### Afuresertib (PKB115125)

AKT is a protein kinase central to many of the signaling pathways, which guide cellular proliferation and apoptosis. Elevated expression of AKT has been demonstrated in myeloma cell lines as well as in bone marrow aspirates form patients with MM. High levels of activated AKT have been revealed in the plasma cells of myeloma patients, with significantly lower levels found in the plasma cells of patients with smoldering myeloma, and lower levels still in those patients with monoclonal gammopathy of unknown significance. Myeloma cell lines subjected to AKT downregulation via small interfering RNA have demonstrated significantly increased rates of apoptosis. Given these findings, AKT seemed to be a rational target in the treatment of MM. The most notable headway to date has been made with the ATP-competitive AKT inhibitor afuresertib [94]. Multiple phase 1 studies establishing the safety and tolerability of afuresertib, both as monotherapy and in combination with other agents, have already been completed. In a phase 1 trial of 73 patients treated with oral afuresertib, toxicities proved relatively benign with the most frequent adverse events being nausea (35.6 %), diarrhea (32.9 %), and dyspepsia (24.7 %) and the dose-limiting toxicity proving to be liver function test abnormalities found in 2 patients treated at the highest investigated dose [95]. Afuresertib has also demonstrated a favorable safety profile when combined with bortezomib and dexamethasone in patients with relapsed/refractory MM in a phase 1b trial which had enrolled 67 patients at the time of last data cutoff [96]. This same trial has also preliminarily demonstrated the significant clinical activity of the combination with an ORR (based on serum M-protein or free-light-chain levels) approaching 50 % in a population of relapsed/refractory patients [96].

### Bcl-2 inhibitors

#### ABT 199

Bcl-2 and its associated family of proteins are crucial regulators of cell death. Bcl-2 itself is known to be an essential anti-apoptotic protein and a rational target for novel chemotherapeutic agents. Small molecule inhibitors of the Bcl-2 family of proteins have been in the preclinical stages of development for over a decade and, after encouraging results, have been graduating to phase 1 clinical trials. Among the earliest such agents was ABT-737, an inhibitor of the Bcl-2 family proteins Bcl-2, Bcl-xL, and Bcl-w. In vitro studies conducted on malignant cell lines showed ABT-737 to be highly effective in indirectly inducing cancer cell apoptosis, and studies in murine models demonstrated its ability to cause tumor regression and promote survival. These preclinical results were particularly impressive with regard to hematologic malignancies [97]. Small molecule inhibitors of Bcl-2 family proteins, such as navitoclax and obatoclax, have entered early-phase clinical trials, particularly for their potential roles in small cell lung cancer. The Bcl-2-specific agent ABT-199 has been under preclinical investigation for hematologic malignancies with multiple trials currently underway [98]. ABT-199 is the first in-class orally bioavailable Bcl-2-specific small molecule inhibitor to be developed. Ex vivo studies of its use in hematologic malignancies have been encouraging, and unlike other agents in its class (ABT-737, navitoclax), it does not appear to be associated with severe use limiting thrombocytopenia [98]. Preclinical investigations into its utility in MM have suggested therapeutic potential in certain subsets of patients. Myeloma cell lines particularly dependent on Bcl-2 for survival are sensitive to ABT-199, whereas cell lines dependent or co-dependent on other Bcl-2 family proteins are less sensitive to Bcl-2 inhibitors [99]. Interestingly, ABT-199 has demonstrated considerable ex vivo synergy with the novel proteasome inhibitor carfilzomib, ostensibly because among carfilzomib’s manifold mechanisms of action is induction of Noxa, a pro-apoptotic member of the Bcl-2 family [99]. ABT-199 has also been shown to be effective in cell lines of MMs with t(11;14), and accordingly, Bcl-2 may thus be a promising targeted therapy for this particular myeloma subtype [97]. ABT-199 therapy has thus far demonstrated significant therapeutic activity in chronic lymphocytic leukemia and non-Hodgkin lymphomas, and a phase 1 trial in relapsed MM is ongoing [97]. The major dose-limiting toxicity in clinical trials was tumor lysis syndrome (TLS). Preclinical trials demonstrated that ABT-199 can induce rapid and profound apoptosis of CLL cells, sometimes within only 8 h, and similar finding have been demonstrated in vivo. The rate of significant TLS in CLL patients is 5 %, including two deaths and one instance of acute renal failure. Such rapid tumor lysis has not yet been observed in preclinical studies with MM; however, as investigations transition to the clinical phase measures toward tumor lysis prophylaxis may be warranted [100].

### BTK inhibitors

#### Ibrutinib

Bruton’s tyrosine kinase (BTK) is an enzyme that plays a crucial role in B cell survival and maturation. Ibrutinib, an orally administered selective inhibitor of BTK, has demonstrated impressive efficacy in the treatment of B cell malignancies and is FDA approved as a therapy for mantle cell lymphoma and chronic lymphocytic leukemia [101, 102]. Ibrutinib, via the inhibition of BTK, has been shown to interfere with intracellular B cell signaling as well as with malignant B cells’ ability to interact with potentially protective microenvironments. These effects limit the malignant B cells’ survival ability and promote apoptosis [101]. Recent preclinical studies have demonstrated that BTK may also be involved in the propagation and maintenance of malignant plasma cell clones in MM [101, 103]. Expression of BTK in malignant plasma cells is increased fourfold relative to benign controls and comparable to BTK expression in CLL and MCL [104]. BTK expression has been shown to be markedly elevated and activated in dexamethasone-resistant MM cell lines, a shared single nucleotide polymorphism in the BTK gene has been demonstrated in a subset of myeloma cell lines, and patients with higher BTK expression in their malignant plasma cells have been shown to have a worse prognosis [101]. Ibrutinib has been shown to be cytotoxic to malignant plasma cells in vitro, and treatment with ibrutinib has been shown to augment the cytotoxic activity of bortezomib and lenalidomide. Ibrutinib activity in myeloma cells seems to be mediated by its ability to interfere with the NF-kB-signaling pathway and thus promote apoptosis [102]. Ibrutinib is presently being evaluated in a phase 2 dose escalation study, as a single agent or in combination with dexamethasone, in patients with relapsed or refractory MM, with preliminary results demonstrating evidence of anti-tumor activity in a heavily pretreated population [104].

### CDK inhibitors

#### Dinaciclib

Dinaciclib is a novel small molecule inhibitor of cyclin-dependent kinases (CDKs), the ubiquitous protein kinases central to the regulation of the cell cycle. Dinaciclib primarily inhibits CDK1, CDK2, CDK5, and CDK9 [105]. It has proven to be relatively well tolerated in initial phase 1 trials and has demonstrated clinical efficacy in chronic lymphocytic leukemia and solid tumors [105]. Dinaciclib was investigated in a dose escalation trial as a single agent for relapsed/refractory MM. Twenty-seven patients with a median of four prior therapies were treated with dinaciclib as a single agent. Three of the 27 (11 %) patients demonstrated a confirmed PR with 2 of them demonstrating a very good PR, and an additional 2 patients demonstrated a minimal response (clinical benefit rate was 19 %) [105]. CDK5 inhibition has been shown to enhance the activity of proteasome inhibitors in vitro, suggesting that a trial combination of dinaciclib and bortezomib may yield fruitful results in the future [105].

### IL-6 inhibitors

#### Siltuximab

Interleukin 6 has been shown to act as a survival factor and growth factor for MM cells in preclinical studies. Serum IL-6 concentration has been shown to correlate with disease stage and prognosis in MM. Increased concentrations of IL-6 in MM are thought to arise from bone marrow stromal cells, and IL-6 is thought to promote the survival of malignant plasma cells via interactions with adhesion molecules, cytokines, tumor suppressor genes, and oncogenes [106]. Siltuximab, a chimeric monoclonal antibody which binds IL-6, has been approved by the FDA for the treatment of multicentric Castleman’s disease and is being investigated in MM [107]. A phase 2 randomized study comparing a bortezomib-melphalan-prednisone regimen with and without siltuximab in 106 transplant-ineligible patients with newly diagnosed MM was conducted. VGPR rate was significantly improved with siltuximab compared to without (71 vs 51 %, *p* = 0.0382); however, the study failed to confirm its hypothesis that the addition of siltuximab would improve the CR rate by at least 10 % with a CR rate proved to be 27 % with siltuximab versus 22 % without it. ORR was also not significantly improved (88 % with siltuximab compared to 80 % without). Median PFS was 17 months, and 1-year overall survival was 88 % and both were identical in the 2 arms [108]. Another phase 2 study, which was a randomized, double-blind, placebo-controlled trial, was performed comparing siltuximab and bortezomib versus bortezomib alone in patients with relapsed/refractory MM. Two hundred eighty-one patients were randomized, and the study failed to show that the addition of siltuximab to bortezomib yields a significant improvement in PFS, or median overall survival, or ORR, or CR rate [107]. Siltuximab is presently being studied in high-risk smoldering MM and may have a future in that aspect and so far of unclear significant in MM patients.

### Kinesin spindle protein inhibitors

#### Filanesib (ARRY-520)

Kinesin spindle protein (KSP), a constituent of the kinesin class of microtubule-based proteins, plays a key role in centrosome separation and bipolar spindle assembly during mitosis. KSP is also believed to have anti-apoptotic properties via mediation of the cell survival protein myeloid leukemia sequence 1 (Mcl-1). Filanesib is a KSP inhibitor with significant anti-tumor activity. Among filanesib’s mechanisms of action is inhibition of cell proliferation via preclusion of proper mitotic spindle assembly. However, its most robust anti-myeloma properties seem to lie in its ability to promote apoptosis via the degradation of Mcl-1. Proliferating hematopoietic cells, including myeloma cells, are particularly dependent on Mcl-1 and thus particularly sensitive to inhibition of KSP. This principle underlies the study of KSP inhibitors in the treatment of MM [109].

Phase 1 trials of filanesib in relapsed and refractory MM, both as monotherapy and in combination with various agents including dexamethasone, bortezomib, and carfilzomib, have demonstrated its toxicities to be largely hematologic [110, 111]. The dose-limiting toxicity is neutropenia, which often requires granulocyte colony-stimulating factor (G-CSF) support [109]. A number of phase 1 trials demonstrated preliminary indications of filanesib efficacy in MM. In a phase 1 study of filanesib, bortezomib, dexamethasone, and prophylactic G-CSF in relapsed/refractory patients revealed that 4 of 13 (31 %) patients on high-dose filanesib/bortezomib demonstrate a PR with significantly inferior results in the low-dose cohort [110]. In a phase 1 trial of filanesib with carfilzomib in 19 relapsed/refractory patients, the ORR was 58 % [111]. A phase 2 study investigating filanesib both with and without low-dose dexamethasone in patients refractory to both bortezomib and lenalidomide showed that among the 32 patients treated with filanesib alone, minor response was observed in 19 % and PR was observed in 16 %. Among the 18 patients treated with filanesib and low-dose dexamethasone, the ORR was 28 % with 22 % demonstrating PR or greater [112]. Preclinical studies have demonstrated that the acute phase protein alpha-1 acid glycoprotein (AAG) can bind filanesib, reducing the amount of free drug and possibly its treatment effect [113]. This observation was taken into account in a phase 2 trial of filanesib with and without dexamethasone in relapsed/refractory patients. This study included 2 patient cohorts: one double refractory to both lenalidomide and bortezomib that was treated with single-agent filanesib and another cohort triple refractory to lenalidomide, bortezomib, and dexamethasone that was treated with filanesib in combination with dexamethasone. Baseline plasma AAG levels were measured in both cohorts. Thirty-two patients were enrolled in the double refractory cohort and 50 patients in the triple refractory cohort. Patients in the triple refractory cohort had more prior treatments and shorter times to progression on most recent treatment on average. ORR in both of these heavily pretreated cohorts was 16 %. Of note, patients with high AAG demonstrate an ORR of 0 % and patients with low AAG demonstrated an ORR (across both cohorts) of 24 % [111]. These studies suggest that there may be a role for filanesib in the future in combination therapy for patients with refractory MM and low AAG.

### PI3K inhibitors

The phosphoinositide 3-kinase (PI3K) family of enzymes are a group of lipid kinase signal transducers involved in a diverse array of cellular functions including growth, proliferation, differentiation, and survival. Among the various classes of PI3Ks, class 1 isoforms have proven to be the major target in drug designs. Perifosine, a combined PI3K and AKT inhibitor, was among the first PI3K inhibitors to be investigated in clinical trials. Although it initially displayed promise in the treatment of relapsed and refractory MM, a phase 3 trial comparing perifosine, bortezomib, and dexamethasone to bortezomib and dexamethasone alone was discontinued in 2013 after interim results showed that the addition of perifosine had not, and likely would not, significantly extend PFS. Another PI3K inhibitor, idelalisib, has proven more successful in the treatment of hematologic malignancies. In 2014, idelalisib was approved for the treatment of relapsed chronic lymphocytic leukemia (CLL), small lymphocytic lymphoma (SLL), and follicular lymphoma (FL) [114].

Numerous PI3K inhibitors are presently undergoing preclinical investigation for their potential use in MM. BAY80-6946, an inhibitor of PI3K-a has demonstrated significant, dose-dependent, anti-tumoral effects across a number of MM cell lines where it was shown to promote cell cycle arrest and apoptosis [115]. Notably, BAY80-6946 was shown to interfere with the oncogenic effects of insulin growth factor 1 (IGF-1). Another PI3K inhibitor, GDC-0941, has also been shown to induce cell cycle arrest and apoptosis in myeloma cell lines [116]. It has shown significant synergy with lenalidomide and dexamethasone in murine xenograft tumor models. Buparlisib, an oral PI3K inhibitor with great promise in breast cancer and other solid malignancies, has also demonstrated encouraging results in mouse models of myeloma and, uniquely, has been shown to mediate a reduction in osteolytic lesions via downregulation of osteoclasts and upregulation of osteoblasts [117]. We must await further studies to determine the potential of PI3K inhibitors in the treatment of relapsed/refractory MM; however, based on the preclinical data to date, and their success in other malignancies, there seems to be a future for their use in MM.

## The role of novel agents in emerging treatment paradigms

A decade ago, the introduction of bortezomib and lenalidomide revolutionized the therapeutic approach to MM and established a new front line for treatment of the disease. Today, these agents remain at the backbone of initial treatment regimens such as cyclophosphamide/bortezomib/dexamethasone (CyBorD) and lenalidomide/bortezomib/dexamethasone (RVD) [26, 27]. Nevertheless, in spite of the advancements offered by bortezomib and lenalidomide, the natural course of MM often remains one of relapse and progressive refractoriness [33]. Although bortezomib- and lenalidomide-based regimens have proven effective in relapsed disease, repeatedly relapsed or high-risk clones can and do become refractory to one or both of these agents [34, 35]. There is thus great interest in developing new agents which remain effective in patients refractory to conventional therapy, agents which may be tapped once the front line has been exhausted. There have been six such agents approved for use in relapsed/refractory MM since 2012, four of which have been approved since 2015.

Among the most promising of these agents include novel proteasome inhibitors and immunomodulators which intuitively build upon the progress made by their predecessors, bortezomib and lenalidomide, respectively. The first of this new generation of agents to receive FDA approval was the novel immunomodulator pomalidomide (Pomalyst) which demonstrated impressive efficacy in cases refractory to lenalidomide, bortezomib, or both [37, 38, 42]. As such pomalidomide is approved for use, alone or in combination with dexamethasone, in relapsed/refractory MM patients who have received at least two prior therapies including lenalidomide and bortezomib and have demonstrated disease progression within 60 days of their most recent treatment [41]. The approval of pomalidomide was closely followed by the approval of two novel proteasome inhibitors, carfilzomib (Kyprolis) and ixazomib (Ninlaro). On the strength of the ASPIRE trial, carfilzomib (in combination with lenalidomide and dexamethasone) gained approval for the treatment of patients with relapsed MM who have received one to three prior lines of therapy [45]. The effectiveness of carfilzomib was underscored by the ENDEAVOR trial in which carfilzomib demonstrated clear clinical superiority to its predecessor bortezomib with regard to the primary endpoint of PFS [45, 47]. Ixazomib earned its approval largely on the strength of the TOURMALINE-MM1 trial where it demonstrated significant efficacy in combination with lenalidomide and dexamethasone [54]. Ixazomib is approved (in combination with lenalidomide and dexamethasone) to treat patients who have received at least one prior therapy. Pomalidomide, carfilzomib, and ixazomib offer effective options for relapsed patients who have developed refractoriness to lenalidomide and bortezomib. For the time being, these novel agents remain “second line”; however, trials such as ENDEAVOR suggest that they may soon play a role on the front line of treatment, especially among high-risk patients. The most effective combinations of these agents among each other, with other novel agents, and with established frontline agents, are yet to be determined and will likely be the subject of numerous forthcoming trials.

Histone deacetylase inhibitors offer a novel mechanism for MM therapy. Panobinostat (Farydak) remains the first and only drug in this class to have received FDA approval. Largely on the strength of the PANORAMA trials, panobinostat (in combination with bortezomib and dexamethasone) has been approved for the treatment of patients who have received at least two prior standard therapies (including bortezomib and an immunomodulatory agent) [76, 77]. Panobinostat is an effective agent for the treatment of relapsed/refractory disease, and its novel mechanism of action makes it a promising component in combination regimens. To date, it is only approved for combination use with bortezomib; however, we expect future trials to establish its utility in combination with immunomodulators and other novel agents especially carfilzomib.

Among the most exciting areas of progress in myeloma therapy is the development of novel monoclonal antibodies, two of which are now approved for use in relapsed/refractory disease. Daratumumab (Darzalex), a monoclonal antibody targeting CD38, recently received accelerated approval as monotherapy for use in patients who have received at least three prior lines of treatment [67]. In the same month, elotuzumab (Empliciti), a monoclonal antibody targeting the cell surface glycoprotein CS1, received approval for use (in combination with lenalidomide and dexamethasone) in patients who have received one to three prior therapies. Elotuzumab was granted breakthrough therapy designation largely on the strength of the ELOQUENT 2 trial [65, 69]. Daratumumab and elotuzumab signal the emergence of monoclonal antibodies as central agents in the treatment of MM, and numerous other antibodies with various targets are present in the pipeline. As with the above agents, the role of these monoclonal antibodies remains limited to relapsed/refractory disease, and the most effective combination regimens remain to be established.

The emergence of this new generation of novel agents offers many options for the treatment of relapsed/refractory MM. Cases refractory to lenalidomide, bortezomib, or both can now be treated in several possible ways such as with novel immunomodulators, novel proteasome inhibitors, HDCA inhibitors, or monoclonal antibodies. The choice of which novel agent to use is not yet subject to a rigid paradigm and is decided on a case by case basis, with thought given to the nature of the preceding line(s) of treatment, availability, cost, and tolerability. As the optimal combinations of these regimens are not yet established, enrollment in clinical trials, where many of these agents are being evaluated as components of novel regimens, will be the future to pave the way to know the best effective treatment.

## Conclusions

We are in a new era in the treatment of MM where we have newer and better agents than we did just 1 year ago. We have at our disposable more effective treatments and a plethora of novel weapons to target even the toughest relapsed/refractory cases. Successful outcomes are more likely to be achieved with combinations of established agents and the novel agents discussed in this review (Table 1). The future of MM treatment is encouraging and promises better response rates and improved survival through the implementation of novel and rationally designed therapies.

**Table 1:** Novel agents in the treatment of multiple myeloma

Category	Agent	Stage of development	Major trials (All trials in relapsed/refractory pts unless otherwise stated) Major trials (All trials in relapsed/refractory pts unless otherwise stated)	Adverse events (Grade 3/4 only unless otherwise stated) Adverse events (Grade 3/4 only unless otherwise stated)
AKT inhibitors	Afuresertib	Phase 1 clinical trials	*Afuresertib monotherapy in 34 pts, PR 9 %, MR 9 % [95]	*Nausea (35.6 %), diarrhea (32.9 %), dyspepsia (24.7 %) [95]
Alkylating agents	Bendamustine	Phase 2 clinical trials	*Bendamustine-lenalidomide-dexamethasone in 29 pts, 1-year OS 93 %, 1-year PFS 20 % [90] *Bendamustine-bortezomib-dexamethasone in 79 pts, OR 60.8 %. PFS 9.7 months, OS 25.6 months [91] *Bendamustine-lenalidomide-dexamethasone in 29 pts, 1-year OS 93 %, 1-year PFS 20 % [90] *Bendamustine-bortezomib-dexamethasone in 79 pts, OR 60.8 %. PFS 9.7 months, OS 25.6 months [91]	*Neutropenia (62 %), thrombocytopenia (38 %), leukopenia (38 %) [90] *Thrombocytopenia (38 %), infections (23 %), polyneuropathy (grade 4) (7 %), polyneuropathy (52 %) [91] *Neutropenia (62 %), thrombocytopenia (38 %), leukopenia (38 %) [90] *Thrombocytopenia (38 %), infections (23 %), polyneuropathy (grade 4) (7 %), polyneuropathy (52 %) [91]
Bcl-2 inhibitors	ABT 199	Preclinical studies	N/A	N/A
BTK inhibitors	Ibrutinib	Phase 1/2 clinical trials	*Ibrutinib single agent or in combination with dexamethasone, trial ongoing [104]	*Trial ongoing [104]
CDK inhibitors	Dinaciclib	Phase 1/2 clinical trials	*Dinaciclib monotherapy in 27 pts, PR 11 %, CBR 19 % [105]	*Diarrhea (87 %), fatigue (67 %), neutropenia (27 %) [105]
Histone deacetylase inhibitors	Panobinostat	Phase 3 clinical trials Postmarketing surveillance Phase 3 clinical trials Postmarketing surveillance	*PANORAMA 2: panobinostat-bortezomib-dexamethasone in 55 pts, OR 34.5 %, CBR 52.7 %, PFS 5.4 months [76] *PANORAMA 1: 768 pts randomized to bortezomib-dexamethasone with either panobinostat or placebo. PFS panobinostat group 12 months, PFS placebo group 8.1 months (HR 0.63) [77] *PANORAMA 2: panobinostat-bortezomib-dexamethasone in 55 pts, OR 34.5 %, CBR 52.7 %, PFS 5.4 months [76] *PANORAMA 1: 768 pts randomized to bortezomib-dexamethasone with either panobinostat or placebo. PFS panobinostat group 12 months, PFS placebo group 8.1 months (HR 0.63) [77]	*Thrombocytopenia (64 %), fatigue (20 %), diarrhea (20 %) [76] *Thrombocytopenia (67 vs 31 %), neutropenia (35 vs 11 %), diarrhea (26 vs 8 %) [77] *Thrombocytopenia (64 %), fatigue (20 %), diarrhea (20 %) [76] *Thrombocytopenia (67 vs 31 %), neutropenia (35 vs 11 %), diarrhea (26 vs 8 %) [77]
	Ricolinostat	Preclinical studies	N/A	N/A
	Vorinostat	Phase 3 clinical trials	*VANTAGE 095: vorinostat-bortezomib in 143 pts (all bortezomib refractory), OR 17 %, CBR 31 % [85] *VANTAGE 088: 637 pts randomized to bortezomib-vorinostat or bortezomib-placebo. PFS vorinostat group 7.63 months, PFS placebo group 6.83 months, *p* = 0.01 [81] *VANTAGE 095: vorinostat-bortezomib in 143 pts (all bortezomib refractory), OR 17 %, CBR 31 % [85] *VANTAGE 088: 637 pts randomized to bortezomib-vorinostat or bortezomib-placebo. PFS vorinostat group 7.63 months, PFS placebo group 6.83 months, *p* = 0.01 [81]	*Thrombocytopenia (67 %), anemia (38 %), neutropenia (32 %) [85] *Thrombocytopenia (45 vs 24 %), neutropenia (28 vs 25 %), anemia (17 vs 13 %) [81] *Thrombocytopenia (67 %), anemia (38 %), neutropenia (32 %) [85] *Thrombocytopenia (45 vs 24 %), neutropenia (28 vs 25 %), anemia (17 vs 13 %) [81]
IL-6 inhibitors	Siltuximab	Phase 2 clinical trials	*106 pts randomized to bortezomib-melphalan-prednisone with vs without siltuximab. OR 88 vs 80 %, VGPR 71 vs 51 %, (*p* = 0.0382). Median PFS (17 months) and 1-year OS (88 %) identical across both arms [108] *281 pts randomized to bortezomib with siltuximab vs placebo. PFS 8.0 vs 7.6 months (HR 0.869, *p* = 0.345). OR 55 VS 47 % (*p* = 0.213) [107] *106 pts randomized to bortezomib-melphalan-prednisone with vs without siltuximab. OR 88 vs 80 %, VGPR 71 vs 51 %, (*p* = 0.0382). Median PFS (17 months) and 1-year OS (88 %) identical across both arms [108] *281 pts randomized to bortezomib with siltuximab vs placebo. PFS 8.0 vs 7.6 months (HR 0.869, *p* = 0.345). OR 55 VS 47 % (*p* = 0.213) [107]	*Neutropenia (62 vs 43 %), thrombocytopenia (44 vs 25 %), pneumonia (17 vs 17 %) [108] *Neutropenia (49 vs 29 %), thrombocytopenia (48 vs 34 %), all-grade infections (62 vs 49 %) [107] *Neutropenia (62 vs 43 %), thrombocytopenia (44 vs 25 %), pneumonia (17 vs 17 %) [108] *Neutropenia (49 vs 29 %), thrombocytopenia (48 vs 34 %), all-grade infections (62 vs 49 %) [107]
Immunomodulators	Pomalidomide	Phase 2 clinical trials Postmarketing surveillance Phase 2 clinical trials Postmarketing surveillance	*Pomalidomide and dexamethasone in 60 pts. OR 63 %, PFS 11.6 months [37] *Pomalidomide and dexamethasone in 84 pts. OR 35 %, OS 14.9 months, 18-month OS 44 % [39] *Pomalidomide and dexamethasone in 60 pts. OR 63 %, PFS 11.6 months [37] *Pomalidomide and dexamethasone in 84 pts. OR 35 %, OS 14.9 months, 18-month OS 44 % [39]	*Neutropenia (32 %), anemia (5 %), thromboembolism (2 %) [37] *Neutropenia (62 %), anemia (36 %), infections (23 %) [39] *Neutropenia (32 %), anemia (5 %), thromboembolism (2 %) [37] *Neutropenia (62 %), anemia (36 %), infections (23 %) [39]
KSP inhibitors	Filanesib	Phase 2 clinical trials	*Filanesib with and without low-dose dexamethasone in 82 pts. OR 16 % in both cohorts. Among pts with high and low serum AAG, OR was 0 % and 24 % respectively across both cohorts [111]	*Thrombocytopenia (44 vs 42 %), anemia (38 vs 50 %), neutropenia (38 vs 38 %) [111]
Monoclonal antibodies	Daratumumab	Phase 2 clinical trials Postmarketing surveillance	*Daratumumab monotherapy in 106 pts. OR 29.2 %, 1-year OS 65 % [65]	*Anemia (33.0 %), thrombocytopenia (26 %), neutropenia (22.6 %) [65]
	Elotuzumab	Phase 3 clinical trials Postmarketing Surveillance	*ELOQUENT 2: 646 pts randomized to lenalidomide-dexamethasone with and without elotuzumab. OR 79 vs 66 %(*p* < 0.001). PFS 19.4 vs 14.8 months, HR 0.70 (CI 0.57 to 0.85, *p* < 0.001) [70]	*Lymphocytopenia (77 vs 49 %), anemia (19 vs 21 %), thrombocytopenia (19 vs 20 %) [70]
	Indatuximab	Phase 1/2 clinical trials	*Indatuximab-lenalidomide-dexamethasone in 15 pts. OR 78 % [72]	*Hypokalemia, fatigue, diarrhea reported as “most common adverse events” [72]
	SAR650984	Phase 1 clinical trials	*SAR650984 monotherapy in 35 pts. ORR 33 %, CR 11 % [73] *SAR650984 and lenalidomide in 31 pts. ORR 64.5 %, CBR 71 % [74]	*Pneumonia 9 % [73] *Fatigue (41.9 %), nausea (38.7 %), upper respiratory tract infection (38.7 %), and diarrhea (35.5 %) [74]
PI3K inhibitors	Numerous agents	Preclinical studies	N/A	N/A
Proteasome inhibitors	Carfilzomib	Phase 3 clinical trials Postmarketing surveillance	*ASPIRE: 792 pts randomized to lenalidomide-dexamethasone with and without carfilzomib. PFS 26.3 vs 17.6 months, HR 0.69, *p* = 0.0001. OR 87.1 vs 66.7 %, CR 31.8 vs 9.3 % [45] *ENDEAVOR: 929 pts randomized to carfilzomib/dexamethasone vs bortezomib/dexamethasone. PFS 18.7 vs 9.4 months, HR 0.53, CI 0.44 - 0.65, *p* = 0.0001. Complete results pending [46], [47]	*Hypokalemia (9.4 vs 4.9 %), fatigue (7.7 vs 6.4 %), hypertension (4.3 vs 1.8 %) [45] *Adverse event data pending [46, 47]
	Ixazomib	Phase 3 clinical trials Postmarketing surveillance	*TOURMALINE-MM1:722 pts randomized to lenalidomide and dexamethasone with and without ixazomib. PFS 20.6 vs 14.7 months (*p* = 0.012), OR 78 % (median duration 21 months) vs 72 % (median duration 15 months) [54]	*“Most common events” included neutropenia, anemia, thrombocytopenia, and pneumonia [54]
	Marizomib	Phase 1 clinical trials	*Marizomib monotherapy in 15 pts. PR 20 %, CBR 57 % [56]	*Fatigue, gastrointestinal AEs, dizziness, and headache reported as “most common adverse events” [56]
	Oprozomib	Phase 1 clinical trials	*Oprozomib-dexamethasone in 29 pts. OR 33.3 %, CBR 46.7 % [59]	*Diarrhea (38 %), vomiting (19 %), thrombocytopenia (10 %) [59]
